# Nursing students’ work readiness and its influencing factors before participating in a nurse residency program: a multicenter cross-sectional study

**DOI:** 10.3389/fmed.2024.1391907

**Published:** 2024-07-17

**Authors:** Liping Chen, Qin Lin, Ye Xu, Liping Wu

**Affiliations:** ^1^Department of Endocrinology, Chongqing Medical University Affiliated Children's Hospital, Chongqing, China; ^2^National Clinical Research Center for Child Health and Disorders, Chongqing, China; ^3^Ministry of Education Key Laboratory of Child Development and Disorders, Chongqing, China; ^4^Chongqing Key Laboratory of Pediatric Metabolism and Inflammatory Diseases, Chongqing, China; ^5^School of Nursing, Chongqing Medical University, Chongqing, China; ^6^Department of Radiology, Children’s Hospital of Chongqing Medical University, Chongqing, China; ^7^Department of Nursing, Children’s Hospital of Chongqing Medical University, Chongqing, China; ^8^Jiangxi Hospital Affiliated Children’s Hospital of Chongqing Medical University, Chongqing, China

**Keywords:** nurse residency program, work readiness, emotional intelligence, China, influencing factors, survey

## Abstract

**Background:**

Studies have shown that work readiness is closely related to nurses’ role adaptation, career development, and patient safety. However, we know little about the nursing students’ work readiness and its influencing factors before participating in a nurse residency program, and whether factors have changed from before.

**Aim:**

(1) To investigate the work readiness of nursing students ready to engage in a nurse residency program; (2) to identify the factors affecting the nursing students’ work readiness and the associations between emotional intelligence and work readiness; and (3) to discuss the changes of factors affecting nursing students’ work readiness.

**Design:**

An online, multicenter cross-sectional study.

**Methods:**

878 nursing students from eight tertiary hospitals in Chongqing, China were recruited. The online investigation used *the General Information Questionnaire*, *the Nursing Students’ Work Readiness Scale*, and *the Emotional Intelligence scale*. The data were analyzed using IBM SPSS V23.0.

**Results:**

768 valid questionnaires were included in this study. The nurses obtained a work readiness score of 277.08 ± 44.39 and an emotional intelligence score of 89.57 ± 13.89. Univariate analysis revealed that the following factors affected work readiness: age, sex, family support for becoming a nurse, voluntary choice of nursing major, previous experience as a student cadre, scholarship recipient status, willingness to engage in nursing work during the COVID-19 pandemic and confidence in clinical nursing practice. Frequent incidents of violence, poor salary for nurses for the nurse residency program, and low social acceptance were the top three reasons for decreased confidence in clinical nursing among nursing students. Furthermore, multiple linear regression analysis indicated that age, voluntary choice of nursing major, student leadership experience, confidence in clinical nursing work, self-emotion, and emotional application significantly influenced nurses’ work readiness.

**Conclusion:**

Clinical instructors and administrators should dynamically assess nurses’ work readiness, prioritize individuals aged ≤23, who have chosen the nursing profession involuntarily, lack prior experience as student cadres, and exhibit low confidence in clinical nursing work. This focus will enhance their emotional self-management skills and ability to apply emotions effectively, improving their work readiness and training efficacy.

## Introduction

The nursing residency program, a pivotal link between academic and clinical practice, delivers consistent professional education to nursing students after the completion of their foundational courses. Its implementation facilitates students’ adaptation to clinical settings (1, 2). In China, the nurse residency program began in the late 20th century, and the official document “Training Outline for Newly Entered Nurses (Trial)” was issued in 2016 (3). Currently, over 90% of tertiary hospitals in China have adopted a nurse residency program (4), with most hospitals requiring 1–3 years of training experience for new nurse staff recruitment (5).

China’s transition to an aging society has led to a steady increase in patients with chronic diseases, creating an escalating demand for high-quality medical care services. Despite the growth in nursing staff from 4.392 million to 5.209 million in the past 5 years, a significant gap remains (6). As of 2022, China has only 3.71 nurses per 1,000 people (7), falling short of the World Health Organization (WHO) requirement of 4.45 nurses per 1,000 people. In contrast, developed countries like Australia, Japan, and Canada reported ratios of 12.6, 11.2, and 9.1 nurses per 1,000 population, respectively, in 2018 (8). However, the primary factor contributing to the nurse shortage in China appears to be nursing students opting to pursue alternative professions upon graduation and the attrition of existing nurses. Reports indicate that only 6.15% of nursing students enrolled in residency programs express a steadfast commitment to their career choice (9), with a high turnover intention rate of 74.4% during their first year (10, 11). Further investigation reveals that resignations primarily result from inadequate work readiness, job burnout, psychological pressure, and related factors. Hence, it is evident that facilitating nursing students’ workplace preparation, emotional regulation, and adjustment to clinical nursing practice is crucial.

Work readiness refers to the extent to which graduate nurses possess qualities and attributes that prepare them with necessary capacities for achieving success in their professional environment. It influences the transition of nursing students’ role from school to the clinical settings and offers them potentials for career development (12, 13). Based on the Work Readiness Scale developed by Caballero et al., Walker conducted qualitative research to define the connotation of work readiness among graduate nurses, wherein nine items were added and several other items were deleted (14). Additionally, a reliability and validity test was performed. The efforts resulted in the production of the Graduate Nurse Work Readiness Scale (WRS-GN) (15). The scale allows the measurement of graduate nurses’ aptitudes in adapting to their upcoming positions. The measurement encompasses four dimensions: social intelligence, organizational acumen, work competence and personal work characteristics. Social intelligence reflects the ability to effectively communicate with others, collaborate within a team setting, manage interpersonal conflicts, and seek support when needed. Organizational acumen evaluates nurses’ comprehension of ward knowledge, administrative systems and procedures, as well as their enthusiasm for engaging in professional development opportunities. Work competence focuses on assessing clinical skills, theoretical knowledge proficiency, work experience relevance and depth, self-assurance in job performance, and sense of responsibility. Lastly, personal work characteristics delve into examining nurses’ mental resilience levels along with their adaptability and stress management capabilities. Based upon Walker’s work, Li et al. further developed a Chinese version of the WRS-GN (16). This Chinese version provides a handy tool for multi-dimensional comprehensive assessment of the graduate nurses’ work readiness in China.

Situated in Southwest China, Chongqing, one of the four municipalities directly under the Central Government of China, has concentrated resources, a large population, strong economic vitality, and relatively rich employment, education, medical, and other resources. It is also among the southwest region’s early implementers of nursing residency programs. Nevertheless, the transition from student to registered nurse can be complicated and problematic. Previous researches have shown that nurses face a series of problems during the period of nurse residency program including excessive psychological stress, inadequate preparation for clinical settings, poor role adaptation, physical exhaustion, difficulties in handling interpersonal relationships at work, ineffective communication with colleagues or patients, and high incidence of adverse events, etc. These problems have induced an escalation in nurses’ propensity to abandon their posts, while they have also emerged as potential factors that would imperil patient safety (17–21). Among these problems, some, such as inadequate preparation for clinical settings, poor role adaptation, physical exhaustion, difficulties in handling with interpersonal relationships at work, and deficient communication with colleagues and patients, are deemed to be suboptimal work readiness. In order to understand the factors that influence the work readiness of newly graduated nurses in China, we further reviewed the Chinese and English literature on this topic from the past 10 years. According to these studies (16, 22–28), factors influencing the work readiness encompass self-motivated career choice in nursing, experience as a student leader, part-time employment history, familial support, interpersonal skills, educational background and school environment, age demographics, clinical teaching practices, sense of belongingness within the profession, as well as professional knowledge and skills. However, only eight original studies have explored work readiness among new nurses in China, with the earliest one published in 2020. This indicates that research in this area is still at an early stage; and the influencing factors identified vary across the studies. Furthermore, these studies primarily focused on external factors such as educational background, age, knowledge acquisition, clinical skills, etc., but somewhat overlook the potential impact of subjective emotions and mindset on human behaviors. However, according to the Mindsponge theory, the establishment of a new set of attitudes, values, beliefs, and behaviors among individuals is achieved through the intricate interplay between their mindset and environment (29). Therefore, besides investigating the effects of external factors on nursing students’ work readiness, this study will also consider the influence of emotional aspects.

The Mindsponge theory, proposed by Vuong et al., holds that the human brain is like a sponge, absorbing ideas that match an individual’s perspective and squeezing out those that do not, and that mindset seriously affects a person’s perceptions, attitudes, and behaviors. The theory tries to explain the mechanism underlying brain information processing and updating, and has gained extensive use in social psychology research (29–31). According to this theory, we recognized that the development of a “clinical nurse” identity is a gradual and continuous cognitive process, wherein nursing students learn to adapt for clinical work through assimilating or rejecting information and values. Within this dynamic progression, the degree to which nursing students choose to embrace or discard such information and values determines the efficacy of their identity transformation. Therefore, based on the Mindsponge theory, we established the questions about nursing students’ work readiness for a nurse residency program as follows: What is the current status of work readiness among the nurse students undergoing training in Chongqing? What factors actually predict the outcomes of work readiness at present? Have there been any changes in the factors influencing nurse students’ work readiness before participating in a nurse residency program? By a cross-sectional study and review other authors’ findings, we can gain a deeper understanding of the above-mentioned questions, which would help design targeted measures to improve the outcomes of work readiness while to promote a high-quality development of nurse residency programs.

## Methods

### Study design

This online cross-sectional survey was conducted among nurses about to commence a residency program in hospitals in Chongqing, China, from July to October 2023.

### Participants and sample size

All participants were nursing students. The participants were informed that they could voluntarily withdraw from the study at any time. Nursing students recruited in 2023 for a nurse residency program at eight tertiary hospitals in the main urban area of Chongqing, China, were enrolled. Inclusion criteria included: (1) newly recruited for a nurse residency program in 2023, (2) completion of theoretical and internship studies in nursing, and (3) agreement to participate. Following Kendall’s sample size estimation method, the minimum sample size was 5–10 times the number of entries (32). Considering a 20% loss rate, the formula for calculating the sample size was as follows: number of questionnaire entries × (5–10) × (1 + 20%). This study included 53 entries, indicating that the minimum sample size ranged from 318 to 636, as calculated using the formula 53 × (5–10) × (1 + 20%).

### Questionnaires

*The General Information Questionnaire* (Table 1), includes age, sex, education level, residence, voluntary choice of nursing profession, and confidence in nursing work.

**Table 1:** General information about the nurses and the candidate factors influencing their work readiness.

Category	Options	*N* (%)	Score (x ± s)	*F*/*t*	*p*-value
Age	≤23 years old	527(68.6)	274.48 ± 42.95	2.406^1^	0.016
	>23 years old	241(31.4)	282.76 ± 46.99		
Sex	Male	75(9.8)	290.93 ± 43.33	2.859^1^	0.004
	Female	693(90.2)	275.58 ± 44.27		
Place of Residence	Urban	411(53.5)	278.14 ± 43.03	0.709^1^	0.479
	Rural	357(46.5)	275.86 ± 45.94		
Only child	Yes	205(26.7)	276.74 ± 46.38	−0.127^1^	0.899
	No	563(73.3)	277.20 ± 43.68		
Fresh graduates	Yes	454(59.1)	275.85 ± 43.86	−0.920^1^	0.358
	No	314(40.9)	278.86 ± 45.23		
Educational background	Junior college or below	442(57.6)	279.26 ± 45.39	1.588^1^	0.113
	Bachelor degree or above	326(42.4)	274.12 ± 42.89		
Whether any immediate family members are nurses	Yes	57(7.4)	284.19 ± 49.28	0.098^1^	0.209
	No	711(92.6)	276.51 ± 43.96		
Whether your parents support you becoming a nurse	Yes	698(90.9)	279.66 ± 43.46	13.438^2^	<0.001
	No	10(1.3)	247.50 ± 43.55		
	Not sure	60(7.8)	251.95 ± 46.34		
Whether you choose the nursing profession voluntarily	Yes	662(86.2)	282.23 ± 41.51	7.529^1^	<0.001
	No	106(13.8)	244.88 ± 48.31		
Have you ever served as a student leader	Yes	514(66.9)	281.96 ± 43.81	4.384^1^	<0.001
	No	254(33.1)	267.21 ± 43.99		
Have you ever received a scholarship	Yes	322(41.9)	281.50 ± 44.43	2.353^1^	0.019
	No	446(58.1)	273.89 ± 44.14		
Are you confident in clinical nursing practice	Yes	600(78.1)	286.38 ± 40.01	75.576^2^	<0.001
	No	53(6.9)	231.32 ± 47.18		
	Not sure	115(15.0)	249.65 ± 40.56		
Whether your willingness to engage in nursing has increased during the COVID-19 pandemic	Yes	543(70.7)	286.37 ± 41.12	47.015^2^	<0.001
	No	97(12.6)	248.97 ± 49.19		
	Not sure	128(16.7)	258.98 ± 39.39		

1 *t*.

2 *F*.

*The Work Readiness Scale* (33), developed by Walker in 2015 and subsequently translated into Chinese by Li Jiaying et al. in 2019, consists of 37 items encompassing five dimensions: job competitiveness, social ability, career development, organizational acumen, and personal work characteristics (16). A 10-level Likert scale was employed for scoring purposes, where 1 indicated “strongly disagree” and 10 signified “strongly agree.” Items 34–37 were reverse-scored questions aimed at assessing job readiness. Higher scores indicated greater levels of job readiness. Cronbach’s alpha coefficient for this study was 0.967.

*The Emotional Intelligence Scale*, compiled by Wong et al. in 2002, and translated into Chinese by Yefei in 2010 (34), contains 16 items across four dimensions: self-emotion assessment, others’ emotion assessment, emotional application, and emotional control. A seven-level Likert scale was adopted, where a score of 1 represented “strongly disagree” and a score of 7 indicated “strongly agree.” Cronbach’s alpha coefficient for this study was 0.962.

### Ethical considerations

This study was approved by the Institutional Review Board of the Children’s Hospital of Chongqing Medical University (approval number: 2023-468). All participants were required to answer one question before completing the electronic questionnaire: Do you give your informed consent to participate in this study? If they chose “No,” they were transferred to the end of the questionnaire and did not complete the rest of the questionnaire.

### Data collection

Data collection occurred between August and September 2023. The survey purpose was explained to the nurse residency training base management staff, in each surveyed hospital, and obtained their consent. The Wenjuanxing (a mobile APP) hyperlink was sent to eligible nursing students via WeChat or Tencent QQ. Informed consent was obtained from the website upon clicking on the link. Qualified nurses had 2 weeks to complete the questionnaire.

### Bias control

Uniform instructions clarified the study’s purpose, survey content, inclusion and exclusion criteria, and logical control design. Each IP user was allowed to complete the form only once, ensuring anonymity. The form could not be submitted until all the questions were completed. Questionnaires with a filling time of less than 300 s, logically inconsistent answers, and identical options for all questions were excluded after two-person data verification.

### Data analysis

SPSS software (version 23.0) was used for statistical analysis. Descriptive statistics expressed data as proportions, frequencies, and percentages for binary variables (i.e., sex), and mean and standard deviation (SD) for continuous variables (i.e., score). One-way analysis of variance (ANOVA) and independent sample t-tests were used to complete the inferential statistics. Pearson’s correlation analysis and multiple linear regression methods were employed to analyze the factors affecting work readiness scores. Statistical significance was set at *p* < 0.05. Independent variables were assigned as follows: (1) Age: ≤ 23 years old = 0, >23 years old = 1; (2) sex: male = 0, female = 1; (3) whether your immediate family members are engaged in nursing: no = 0, yes = 1; (4) whether your parents support you being a nurse: no = 0, not sure = 1, yes = 2; (5) whether you voluntarily chose the nursing profession: no = 0, yes = 1; (6) have you ever served as a student leader: no = 0, yes = 1; (7) have you ever received a scholarship: no = 0, yes = 1; (8) whether your willingness to engage in nursing has been enhanced during the COVID-19 pandemic: no = 0, not sure = 1, yes = 2; (9) are you confident in clinical nursing: no = 0, not sure = 1, yes = 2.

## Results

### General characteristics

In this study, 878 students completed the questionnaire, and 768 valid responses were collected, resulting in an effective recovery rate of 87.47%. Excluded were questionnaires with answer times <300 s, logical contradictions between the options, and instances where all items selected the same option. The age of the nursing students ranged from 20 to 30 years (mean age, 22.96 ± 1.59). Further general data are presented in Table 1.

### Work readiness scores

The work readiness scores of the nursing students ranged from 115 to 367 (277.08 ± 44.39), with scores in each dimension as follows: job competitiveness (54.95 ± 11.58), social ability (64.01 ± 13.65), career development (70.26 ± 13.34), organizational acumen (59.62 ± 9.87), and personal work characteristics (28.23 ± 5.24). The work readiness scores of nursing students with different social characteristics are shown in Table 1.

### Factors influencing work readiness

**Figure 1:**
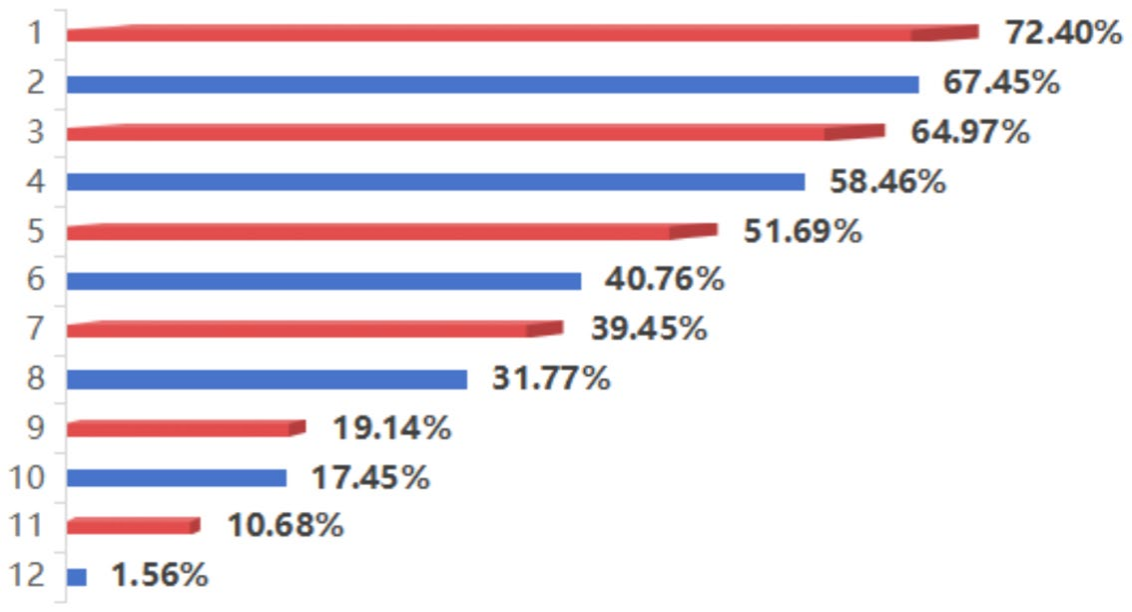
Factors reported by nursing students that reduce their work confidence. (1) Frequent incidents of doctors/nurses being injured by violence; (2) Poor pay and benefits for nurses; (3) Low social recognition of nurses; (4) Face a variety of tests/inspections in the work; (5) The workload is large and the content is tedious; (6) Nurses are required to work midnight shifts; (7) Depressing atmosphere in the internship unit; (8) No space for development in the workplace; (9) Lack of self-competency; (10) The tutor during the internship is irresponsible; (11) There are few practical operation opportunities during the internship; (12) Other else.

One-way analysis revealed eight factors significantly influencing the work readiness of nursing students (*p* < 0.05 for all eight factors), as presented in Table 1. These factors included age, sex, parental support for pursuing nursing, voluntary choice of nursing profession, experience as a student leader, receipt of a scholarship, confidence in clinical nursing, and whether the willingness to engage in nursing had been enhanced during the COVID-19 pandemic. The factors decrease their work confidence were shown in the Figure 1.

### Correlation analysis between emotional intelligence and work readiness

The total scores of the Emotional Intelligence Scale for training nurses ranged from 20 to 110 (89.57 ± 13.89). Scores for each dimension were as follows: self-emotion assessment (22.56 ± 3.77), others’ emotion assessment (22.32 ± 3.63), emotion application (22.42 ± 3.73), and emotion control (22.27 ± 3.91). Pearson’s correlation analysis revealed a significant positive correlation between work readiness and emotional intelligence (r = 0.692, *p* < 0.001). Furthermore, the correlation coefficients between work readiness and the emotional intelligence dimensions were 0.658, 0.580, 0.678, and 0.638, respectively, with a significance level of *p* < 0.001.

### Multiple linear regression analysis of work readiness

The dependent variable in this study was nurses’ work readiness scores. Independent variables included those with statistical significance in both univariate and correlation analyses, while emotional intelligence scores were retained in their original values. Findings indicated that emotion self-assessment, emotion application, confidence in clinical nursing work, experience as a student leader, age, and voluntary choice of nursing major were factors significantly affecting training nurses’ work readiness (*p* < 0.05). These factors account for 51.1% of the observed variations (Table 2).

**Table 2:** Multiple linear regression analysis of the factors influencing nurses’ work readiness.

Variable	Regression coefficient	Standard error	*t*-value	*p*-value
Constant	76.893	7.194	10.688	<0.001
Self-emotion assessment	4.675	0.551	8.486	<0.001
Emotional application	2.968	0.551	5.391	<0.001
Are you confident in clinical nursing practice	9.903	2.210	4.481	<0.001
Have you ever served as a student leader	5.566	2.420	2.300	0.022
Age	4.909	2.433	2.018	0.044
Whether you choose the nursing profession voluntarily	7.193	3.598	1.999	0.046

## Discussion

Following the application of the inclusion and exclusion processes, 768 questionnaires were deemed suitable for analysis. The findings revealed that the work readiness of newly trained nurses in Chongqing was moderate. Their scores slightly surpassed those of new nurses in Henan (272.25 ± 52.20) (25), new nurses in Hubei (268.93 ± 68.64) (26), in-training nurses in Xinjiang (265.72 ± 47.79) (27), and the research results by Li et al., reporting scores of (261.51 ± 45.40) (28). This discrepancy may be attributed to regional and subjective variations within the context of these studies. The scores in this study were marginally higher than those of nursing students who had not completed clinical practice in Saudi Arabia (35). This could be owing to the fact that the training nurses had finished their clinical practice and accumulated certain clinical experience, making them more prepared for the nurse residency program. Studies conducted in Australia (36, 37), the United States (38), and other countries suggest that work readiness among nursing students may evolve over time; however, specific trends remain unclear. Previous researches have consistently demonstrated significant correlations between Chinese nursing students’ work readiness and factors such as their educational background, age, scholarship status, and familial support. Our findings did not reflect such a correlation, suggesting a potential gradual weakening of this factor’s influence. Instead, self-emotional assessment, emotional application ability, fear of violence, and seeking public approval emerge as primary determinants of work readiness. The discrepancy of the findings suggests that the factors affecting the nurse students’ work readiness are changing in China. The reasons remain ambiguous and could be attributed to the dynamic nature of the human mind resulted from variances in societal development and in regional cultural norms, as posited by the Mindsponge theory.

In the present study, older individuals demonstrated higher levels of work readiness, consistent with the findings of Son et al. (25). This could be attributed to their enhanced cognitive abilities and adaptability to various environments. Medical colleges and hospitals should prioritize the evaluation and enhancement of work readiness among younger students through daily teaching practices and pre-service training. Similarly, a study conducted in Saudi Arabia (35) revealed that nursing students who chose nursing majors involuntarily exhibited lower work readiness (27). For these students, nursing is not their primary career choice, resulting in diminished interest, potential resistance, reduced enthusiasm for learning, limited theoretical knowledge, and limited practical skills. Consequently, they lack confidence in clinical nursing practice and exhibit suboptimal readiness for training programs. Therefore, educators and trainers should guide nursing students toward developing a correct understanding of the nursing profession while fostering an improved sense of professional identity. In Chinese universities, class cadres are typically required to assist teachers in completing a range of teaching and class management tasks, effectively enhancing their communication skills and ability to manage various responsibilities. Moreover, this experience enabled them to remain composed when faced with environmental and role changes. Encouraging students’ active participation in class affairs is advisable as it helps cultivate their confidence in clinical nursing work.

At present, violence against medical professionals, including doctors and nurses, is a prevalent issue in China. The perpetrators of such violence are often patients’ family members, Violence from them is closely associated with the low job satisfaction, strong inclination to resign, negative emotions, and burnout experienced by nurses (39, 40). A meta-analysis reveals that approximately 71% of nurses in China have been subjected to some form of violence, primarily consisting of verbal aggression. This percentage is notably higher than that observed in the United States and Europe, which ranges from 54 to 62% (41–43). Violence would probably bring fear to nursing students who have not yet entered the nursing profession and induce them losing confidence to their professions, and reduce their resilience during the school-clinic transition (44). Therefore, we desperately hope the improvement of laws and regulations, the offer of effective preventive and emergency measures, and the reduction of violence affairs, among other measures. In this study, poor salary and welfare benefits was also found to be a major reason resulting in the nurses’ lack of confidence in clinical work. According to the data collected by Guo et al., merely 932 (41.40%) of the 2,251 nurses were content with their salary and benefits (5). In contrast, Yuan et al.’s study revealed a significantly lower proportion (16.82%) (45). Furthermore, a systematic review substantiated that low pay was the precise factor leading to nurses’ declining confidence and subsequent departure (46). In agreement with the findings of these studies, our study also indicates that the salary and welfare of nurses in China require for further improvement. In short, it is essential to establish a rational salary distribution system, thereby bolstering nurses’ confidence and enthusiasm for their work. Public disapproval of nurses is the third leading cause contributing to the lack of confidence in nurses, which can be exacerbated by misleading reporting of medical disputes and stigmatization of medical personnel in China (47). Therefore, we think it would be vital that nurses feel valued and respected by society, given their immeasurable commitment and dedication.

Emotional intelligence refers to an individual’s capacity to comprehend and effectively utilize emotional information to understand both their own emotions and those of others, ultimately leading to problem-solving. This multifaceted construct encompasses four key dimensions: emotional self-assessment, assessment of others’ emotions, emotional application, and emotion control. As a crucial psychological resource, emotional intelligence plays an important role in facilitating positive feedback (48, 49). Nurses who possess high levels of emotional intelligence demonstrate enhanced psychological mastery, adeptly perceive patients’ emotions, effectively manage and regulate their own emotions, and exhibit the ability to promptly adjust their thinking patterns while calmly addressing nurse–patient conflicts. Moreover, they are more inclined to employ coordinated conflict-management strategies that foster harmony by reducing communication barriers and facilitating positive emotional experiences. The findings of this study revealed that the emotional intelligence level of trained nurses was moderate. Self-emotional assessment and emotional application significantly affected the work readiness of training nurses, indicating that higher levels of emotional evaluation, management, and regulation were associated with increased work readiness and greater suitability for clinical nursing practices among nursing students.

The findings of this study indicate that the factors affecting nurses’ work readiness are undergoing changes, educators and managers should dynamically assess nurses’ work readiness, consider individual differences among trained nurses, propose targeted intervention proposals, and implement effective measures to enhance their work readiness. In addition, the nurses’ capacity to effectively manage and apply emotions significantly impacts their level of work readiness. It is imperative for educators and managers in medical institutions to comprehend this aspect and provide support in enhancing students’ emotional cognition, acquiring proficiency in emotional regulation skills, establishing goals for emotional management, as well as fostering an understanding of seeking professional assistance when necessary. Previous studies have demonstrated that emotional intelligence can be improved through appropriate training and interventions (50–53). In the future, integrating emotional intelligence training into nursing humanities courses and vocational training programs in medical institutions could help identify strategies to improve emotional intelligence. For instance, academic lectures, specialized training, and participation in social practice can guide nursing students in developing skills for controlling their own emotions, expressing emotions effectively, and observing others’ emotions to enhance their work readiness (54).

As the famous quote of Louis Pasteur said: “Dans les champs de l’observation le hasard ne favorise que les esprits prepares” (In the fields of observation chance favors only the prepared mind) (55). An efficient work readiness signifies that the graduate nurses would perform better in a nurse residency program and vice versa. In future work, qualitative research should be conducted to further clarify the work experience of nurses in Chongqing and the factors that influence work readiness could not be known through the survey, and to establish a research base for conducting intervention studies. In addition, Harrison et al. have suggested that work readiness begins at the nursing student’s entry into school and ends at the completion of nurse residency program (56). Therefore, medical institutions and educational Institutions should pay special attention to the development of nursing students’ work readiness, strengthen cooperation, and jointly explore the methods to improve nurses’ work readiness, e.g., seeking for the education and training modes, adjusting the nursing curriculum, ensuring sufficient clinical practice opportunities, and helping career role transformation.

## Strengths and limitations

The results of this study underscore the imperative for ongoing enhancement of nurses’ work readiness. The study reveals a correlation between work readiness and several influencing factors, including age, scholarship attainment, voluntary selection of a nursing major, confidence in pursuing nursing, and self-emotion management. It informs nursing educators and managers to implement targeted interventions based on students’ characteristics. The limitations of this study are as follows: first, the nature of this study as an online survey introduces challenges in establishing causality, and self-report bias may be present in cross-sectional studies. Second, the study participants were exclusively recruited from the main urban area of Chongqing in western China. Considering that Chongqing’s university education level aligns with the national average, the findings may not comprehensively represent the situation in other regions.

## Conclusion

The work readiness and emotional intelligence of nursing students prepared to engage in a nursing residency program in Chongqing, China, are at an intermediate level and warrant further enhancement. Several factors, including age, experience as student leader, confidence in nursing practice, voluntary choice of nursing profession, emotional self-assessment, and emotional application, significantly influence the work readiness of training nurses. Consequently, it is imperative for college educators and medical institution managers to focus on younger nursing students who have involuntarily chosen a nursing major, lack prior experience as student cadres, or exhibit limited confidence in their clinical nursing practice. Implementing effective intervention programs to improve emotional management abilities and emotional application skills can contribute to elevated work readiness levels and improved training outcomes.

## Data availability statement

The raw data supporting the conclusions of this article will be made available by the authors, without undue reservation.

## Ethics statement

The studies involving humans were approved by Institutional Review Board of the Children’s Hospital of Chongqing Medical University. The studies were conducted in accordance with the local legislation and institutional requirements. The participants provided their written informed consent to participate in this study.

## Author contributions

LC: Investigation, Writing – original draft. QL: Supervision, Writing – review & editing. YX: Writing – review & editing. LW: Methodology, Project administration, Writing – review & editing.
